# Evaluating the performance of low-frequency variant calling tools for the detection of variants from short-read deep sequencing data

**DOI:** 10.1038/s41598-023-47135-3

**Published:** 2023-11-22

**Authors:** Xudong Xiang, Bowen Lu, Dongyang Song, Jie Li, Kunxian Shu, Dan Pu

**Affiliations:** Chongqing Key Laboratory of Big Data for Bio Intelligence, Chongqing University of Posts and Telecommunications, Chongqing, 400065 China

**Keywords:** Genome informatics, Software, Data processing

## Abstract

Detection of low-frequency variants with high accuracy plays an important role in biomedical research and clinical practice. However, it is challenging to do so with next-generation sequencing (NGS) approaches due to the high error rates of NGS. To accurately distinguish low-level true variants from these errors, many statistical variants calling tools for calling low-frequency variants have been proposed, but a systematic performance comparison of these tools has not yet been performed. Here, we evaluated four raw-reads-based variant callers (SiNVICT, outLyzer, Pisces, and LoFreq) and four UMI-based variant callers (DeepSNVMiner, MAGERI, smCounter2, and UMI-VarCal) considering their capability to call single nucleotide variants (SNVs) with allelic frequency as low as 0.025% in deep sequencing data. We analyzed a total of 54 simulated data with various sequencing depths and variant allele frequencies (VAFs), two reference data, and Horizon Tru-Q sample data. The results showed that the UMI-based callers, except smCounter2, outperformed the raw-reads-based callers regarding detection limit. Sequencing depth had almost no effect on the UMI-based callers but significantly influenced on the raw-reads-based callers. Regardless of the sequencing depth, MAGERI showed the fastest analysis, while smCounter2 consistently took the longest to finish the variant calling process. Overall, DeepSNVMiner and UMI-VarCal performed the best with considerably good sensitivity and precision of 88%, 100%, and 84%, 100%, respectively. In conclusion, the UMI-based callers, except smCounter2, outperformed the raw-reads-based callers in terms of sensitivity and precision. We recommend using DeepSNVMiner and UMI-VarCal for low-frequency variant detection. The results provide important information regarding future directions for reliable low-frequency variant detection and algorithm development, which is critical in genetics-based medical research and clinical applications.

## Introduction

Detecting somatic variants is crucial to precision medicine and is an active area of research. However, variants of clinical interest may be present at a very low frequency (≤ 1%) due to factors such as normal tissue contamination or variants present in a fraction of the tumor cells in tumor samples and non-invasive biopsies^1^. These extremely low-level variants bear great promise for use as biomarkers for the early detection of cancers, monitoring of disease progression and therapeutic responses, and diagnosis, prognosis, and prediction of drug resistance^2,3^.

The advance of next-generation sequencing (NGS) has accelerated precision medicine by enabling the detection of important genetic variants. However, detecting variants at very low allele fractions (≤ 1%) is challenged by errors introduced in many steps of the NGS process, including library preparation, sequencing, and read alignment, making it difficult to identify true variants without generating false positive variant calls^4^. A variety of variant callers are publicly available and usually do a good job of identifying variants. However, as variant allele frequencies (VAFs) fall below 1%, the specificity of these tools suffers greatly as true variants at such low frequencies can be easily confused with sequencing or PCR artifacts. To address this problem, several raw-reads-based variant callers (Table 1) have been developed, such as SiNVICT^5^, outLyzer^6^, Pisces^7^, and LoFreq^8^. SiNVICT applies a Poisson model to identify potential variants. It enables not only to detect single nucleotide variants (SNVs) and insertions and deletions (indels) with variant allele percentages as low as 0.5% but also to perform time series analysis^5^. outLyzer uses the Thompson Tau test to measure the background noise level and then calls variants with non-reference bases above that level. It had the best sensitivity and specificity with a fixed limit of detection for all tools of 1% for SNVs and 2% for indels^6^. Pisces is tuned for amplicon sequencing data and SNV calling is determined by a q-score based on reference and non-reference read counts and a Poisson model^7^. LoFreq calls variants occurring in < 0.05% of a population based on viewing each base as an independent Bernoulli trial with distinct "success" probability, where success is defined as non-reference and the probability is determined by the base quality score^8^. These raw-reads-based variant callers enable us to identify variants with frequencies as low as 0.05%-1% effectively but at the cost of having a high number of false positives^9^.

**Table 1:** A summary of eight low-frequency variant callers used in this study.

Variant caller	Version	Read type	Code	Model	Detection limit (original paper) (%)
SiNVICT	v1.0	Raw-reads-based	C++ & Python	Poisson model	0.5
outLyzer	v3		Python	Noise level estimation	1
Pisces	v5.3.0.0		C#	Poisson model	–
LoFreq	v2.1.5		C & Python & Shell	Bernoulli trial	0.05
UMI-VarCal	v2.5.0	UMI-based	Python	Poisson test	0.3
smCounter2	v2		Python & R	Binomial distribution	0.5
DeepSNVMiner	v1		Perl & R	SAMtools calmd	0.1
MAGERI	v1.1.1		Java & Groovy	Beta-Binomial model	0.1

Unique molecular identifiers (UMIs) have been proposed and used to filter out artifacts. In brief, this technique uses different molecular barcodes to label each target molecule, and treats reads sharing the same UMI as a "read family", which is used to correct amplification and sequencing errors. Within a read family, true variants should be present on both strands of a DNA fragment and appear in all members of a read family pair, while sequencing errors and PCR-introduced errors occurring late in amplification should be present in only one or a few family members^10^. This technique has been proven to be the most effective method for low-frequent variant calling and has been extensively used in a large number of recent studies^11–14^. Several UMI-based variant callers (Table 1) have been proposed, such as DeepSNVMiner^15^, MAGERI^16^, smCounter2^17^, and UMI-VarCal^9^. DeepSNVMiner first generates an initial list of variants using SAMtools calmd and then selects the high-confidence variants with strong UMI support. However, it might produce a lot of false positives due to the lack of a stand bias filter or a homopolymer region length filter^15^. MAGERI builds a consensus read for each UMI group of reads and takes a Beta-binomial modeling approach. It has a theoretical detection limit of 0.1% but suffers in terms of performance as it consumes a lot of memory and is very slow^16^. smCounter2 has good performance with a detection limit of 1%-0.5% as it adopts Beta distribution to model the background error rates and Beta-binomial distribution to model the number of non-reference UMIs^17^. UMI-VarCal applies a Poisson statistical test at every position to determine background error rates at each position. UMI-VarCal enables the detection of low-frequency variants with very high sensitivity and specificity and shows a detection threshold of 0.1%^9^. In conclusion, some UMI-based variant callers are able to achieve a detection limit as low as 0.1% or even lower, but they vary in terms of sensitivity, precision, running speed, and memory consumption. Therefore, it is necessary to evaluate these variant callers in terms of performance, concordance, and time consumption.

Numerous variant callers designed for variants with VAFs > 1% have been developed, and their performance has been comprehensively evaluated in some previous studies^18–33^. In contrast, only a small percentage of studies have compared the evaluation of these variant callers for low-frequency variant calling. For example, David H Spencer et al. assessed the performance of four variant callers, SAMtools, Genome Analysis Toolkit, VarScan2, and SPLINTER, in detecting low-frequency variants^34^. Sandmann S. et al. evaluated eight open-source tools regarding their ability to call SNVs and short indels with VAFs as low as 1% in non-matched next-generation sequencing data: GATK HaplotypeCaller, Platypus, VarScan, LoFreq, FreeBayes, SNVer, SAMtools, and VarDict^32^. However, those studies only examined the efficacy of variant callers designed for variants with VAFs greater than 1% in finding low-frequency variants, not the efficacy of existing low-frequency variant calling tools designed specifically for low-frequency variants^34^. Other studies have also compared the low-frequency variant calling with variant callers designed for variants with VAFs > 1% as in Etienne Muller et al. studies^6^. Furthermore, some studies have only evaluated partial the UMI-based and the raw-reads-based low-frequency variant callers. Vincent Sater et al. have compared two UMI-based low-frequency variant callers, UMI-VarCal and DeepSNVMiner, with the two best raw-reads-based low-frequency variant callers, SiNVICT and outLyzer^9,35^. And in the study of Chang Xu et al., they have evaluated only two UMI-based low-frequency variant callers, smCounter2 and MAGERI, and two raw-reads-based variant callers designed for variants with VAFs > 1%, VarDict, and Mutect2^17^. Anne Bruun Krøigård et al. focused on the performance of nine publicly available somatic variant callers designed for variants with VAFs greater than 1% for the detection of SNVs and small indels^36^. Only one study focused on comprehensive benchmarking low-frequency variant calling tools. Theresa Lüth et al. have evaluated several low-frequency variant callers designed specifically for low-frequency variant calling, but these low-frequency variant callers were proposed for long-read data generated from nanopore sequencing, limiting the low-frequency variant callers proposed for short-read data generated from widely used sequencing platforms such as Illumina^37^. Therefore, it is necessary to comprehensively evaluate publicly available low-frequency variant callers designed for the detection of the low-frequency variants in short-read data.

In this study, we comprehensively evaluated and compared eight variant callers that were developed specifically for low-frequency variant calling, including four raw-reads-based variant callers, SiNVICT, outLyzer, Pisces, and LoFreq, and four UMI-based variant callers, DeepSNVMiner, MAGERI, smCounter2, and UMI-VarCal, using simulated datasets, reference datasets, and Horizon Tru-Q sample data. We aimed to evaluate the performance of these variant callers for low-frequency variant detection and to identify the most precise and efficient tools for low-frequency variant calling. These were assessed according to performance, concordance, and time consumption to provide a useful guideline for reliable variant calling in genetic medical research and clinical application.

## Results

### Variant calling performance comparison of eight low-frequency variant callers at various VAFs

A series of simulated datasets with various VAFs (5%, 2.5%, 1%, 0.5%, 0.25%, 0.1%, 0.05%, and 0.025%) were generated to assess the performance of eight low-frequency variant callers. The number of variants and artifacts reported by eight variant callers at a high sequencing depth of 20000X was summarized in Table 2. All eight tools performed well and called more than 40 true positive variants at a VAF of 2.5%. Among them, outLyzer identified the most true positive variants (50), followed by smCounter2 (49), Pisces (49), SiNVICT (49), LoFreq (48), UMI-VarCal (48), and DeepSNVMiner (44), with MAGERI identifying the fewest (41). Except for smCounter2, the UMI-based callers outperformed the raw-reads-based callers at VAFs of 2.5%, 5%, and 10%, respectively. For example, at a VAF of 0.05%, variants reported by the UMI-based callers, DeepSNVMiner, MAGERI, smCounter2, and UMI-VarCal accounted for 44, 39, 0, 42 out of 50, respectively, while none of the raw-reads-based callers, SiNVICT, outLyzer, Pisces, and LoFreq could found any variants. However, both the UMI-based and the raw-reads-based callers were unable to call variants present with a VAF of 0.025%. Thus, the detection limits of DeepSNVMiner, MAGERI, and UMI-VarCal were down to 0.05%.

**Table 2:** Performance of eight low-frequency variant callers on simulated datasets at various VAFs of 5%, 2.5%, 1%, 0.5%, 0.25%, 0.1%, 0.05%, and 0.025% with a sequencing depth of 20000X.

Variant caller	VAF = 5%					VAF = 2.5%				
	No. of calls ^*a*^	True positive	False positive	Sensitivity (%)	Precision (%)	No. of calls	True positive	False positive	Sensitivity (%)	Precision (%)
DeepSNVMiner	44	44	0	88.00	100.00	44	44	0	88.00	100.00
MAGERI	41	41	0	82.00	100.00	41	41	0	82.00	100.00
smCounter2	50	49	1	98.00	98.00	50	49	1	98.00	98.00
UMI-VarCal	48	48	0	**96.00**	**100.00**	48	48	0	**96.00**	**100.00**
SiNVICT	56	49	7	98.00	87.50	56	49	7	98.00	87.50
outLyzer	52	50	2	**100.00**	**96.15**	52	50	2	**100.00**	**96.15**
Pisces	63	49	14	98.00	77.78	63	49	14	98.00	77.78
LoFreq	91	48	43	96.00	52.75	91	48	43	96.00	52.75

Variant caller	VAF = 1%					VAF = 0.5%				
	No. of calls	True positive	False positive	Sensitivity (%)	Precision (%)	No. of calls	True positive	False positive	Sensitivity (%)	Precision (%)
DeepSNVMiner	44	44	0	88.00	100.00	44	44	0	88.00	100.00
MAGERI	41	41	0	82.00	100.00	41	41	0	82.00	100.00
smCounter2	0	0	0	0.00	0.00	0	0	0	0.00	0.00
UMI-VarCal	48	48	0	**96.00**	**100.00**	48	48	0	**96.00**	**100.00**
SiNVICT	8	1	7	2.00	12.50	7	0	7	0.00	0.00
outLyzer	3	1	2	2.00	33.33	2	0	2	0.00	0.00
Pisces	21	7	14	14.00	33.33	14	0	14	0.00	0.00
LoFreq	88	20	68	40.00	22.73	82	8	74	16.00	9.76

Variant caller	VAF = 0.25%					VAF = 0.1%				
	No. of calls	True positive	False positive	Sensitivity (%)	Precision (%)	No. of calls	True positive	False positive	Sensitivity (%)	Precision (%)
DeepSNVMiner	44	44	0	88.00	100.00	43	43	0	86.00	100.00
MAGERI	40	40	0	80.00	100.00	39	39	0	78.00	100.00
smCounter2	0	0	0	0.00	0.00	0	0	0	0.00	0.00
UMI-VarCal	47	47	0	**94.00**	**100.00**	46	46	0	**92.00**	**100.00**
SiNVICT	8	0	8	0.00	0.00	7	0	7	0.00	0.00
outLyzer	2	0	2	0.00	0.00	2	0	2	0.00	0.00
Pisces	14	0	14	0.00	0.00	14	0	14	0.00	0.00
LoFreq	72	3	69	6.00	4.17	83	0	83	0.00	0.00

Variant caller	VAF = 0.05%					VAF = 0.025%				
	No. of calls	True positive	False positive	Sensitivity (%)	Precision (%)	No. of calls	True positive	False positive	Sensitivity (%)	Precision (%)
DeepSNVMiner	44	44	0	88.00	100.00	0	0	0	0.00	0.00
MAGERI	39	39	0	78.00	100.00	0	0	0	0.00	0.00
smCounter2	0	0	0	0.00	0.00	0	0	0	0.00	0.00
UMI-VarCal	42	42	0	**84.00**	**100.00**	0	0	0	0.00	0.00
SiNVICT	7	0	7	0.00	0.00	7	0	7	0.00	0.00
outLyzer	2	0	2	0.00	0.00	2	0	2	0.00	0.00
Pisces	14	0	14	0.00	0.00	14	0	14	0.00	0.00
LoFreq	76	0	76	0.00	0.00	80	0	80	0.00	0.00

^*a*^number of calls.

Significant values are highlighted in bold.

**Figure 1:**
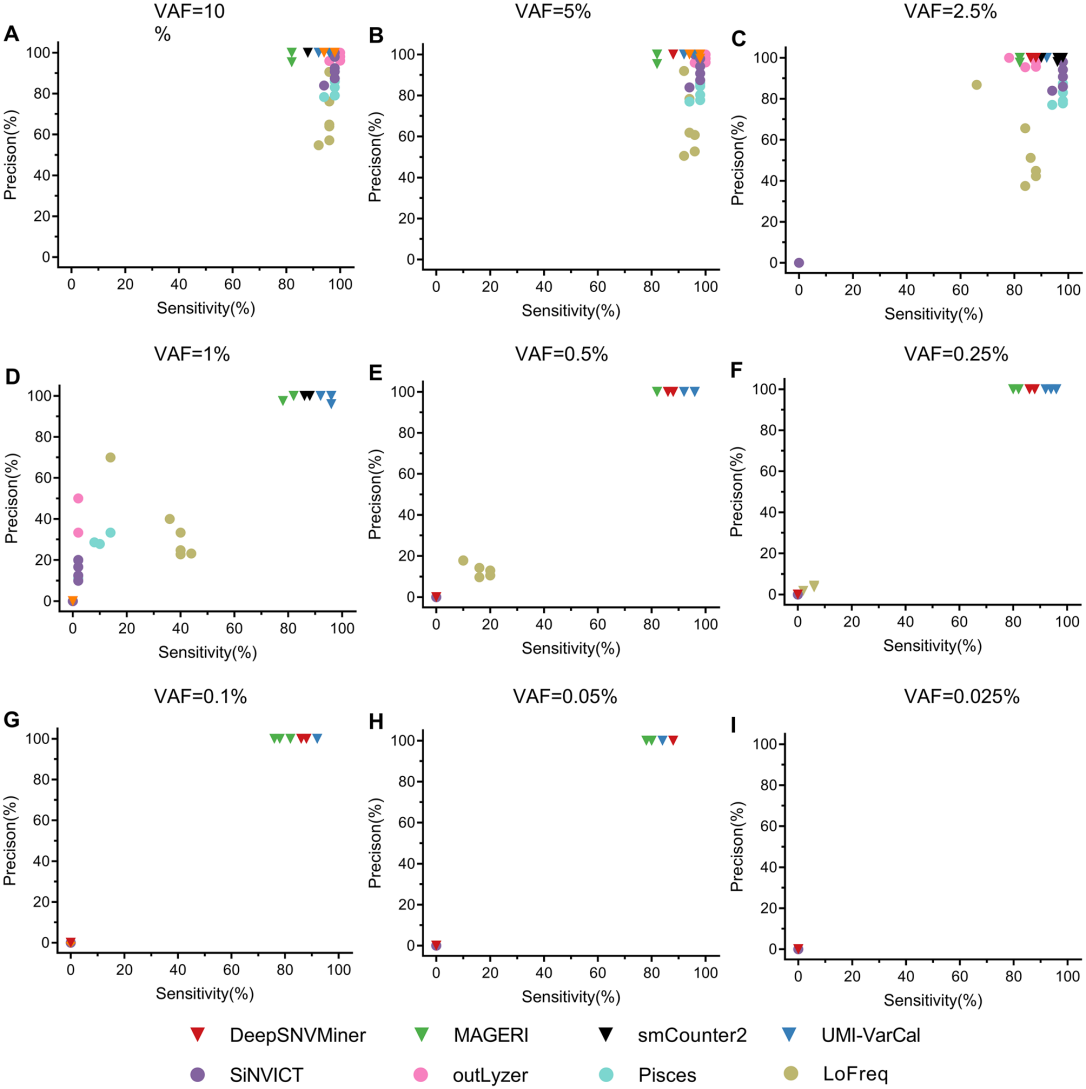
Sensitivity and precision of eight tools on the simulated datasets in case of various VAF levels. VAFs of (**A**) 10%; (**B**) 5%; (**C**) 2.5%; (**D**) 1%; (**E**) 0.5%; (**F**) 0.25%; (**G**) 0.1%; (**H**) 0.05%; and (**I**) 0.025%.

Also, two-dimensional scatter plots of the precision and sensitivity of eight low-frequency callers at various VAFs were displayed in Fig. 1. It was obvious that the sensitivity and precision of each variant caller tended to decrease as the VAFs decreased. Compared to the UMI-based callers, the performance of the raw-reads-based callers decreased even faster. At VAFs of 2.5%, 5%, and 10%, all eight variant callers showed sensitivity and precision ranging from 82 to 100% and 52.75% to 100%, respectively. outLyzer demonstrated the maximum sensitivity (100%), whereas DeepSNVMiner, MAGERI, and UMI-VarCal surpassed the rest in terms of precision. smCounter2, SiNVICT, outLyzer, Pisces, and LoFreq, on the other hand, showed low sensitivity and precision ranging from 0 to 16% and 0% to 9.76% at VAFs of 1% and 0.5%, respectively. At a VAF of 0.25%, UMI-VarCal took the top spot, followed by DeepSNVMiner in second place and MAGERI in third. Additionally, they all exhibited exceptional performance at a VAF of 0.25% or higher with assuredly sensitive and precise callings. At VAFs of 0.05% to 0.1%, DeepSNVMiner, MAGERI, and UMI-VarCal outperformed the other five callers in both sensitivity and precision. They appeared to be the only three tools with good sensitivity (> 75%) and excellent precision (100%) at high sequencing depths (Fig. 1, Supplementary Tables S3–S11). In addition, UMI-VarCal performed the best at VAFs of l% to 0.1% with sensitivity and precision of 92% and 100%, respectively, while DeepSNVMiner (sensitivity and precision of 86% and 100%) and MAGERI (sensitivity and precision of 78% and 100%) came in second and third, respectively. However, at the lowest VAF of 0.025%, regardless of the sequencing depth, none of the eight low-frequency variant callers were able to call any variants. (Fig. 1 and Supplementary Tables S3–S11). All in all, in terms of precision, the UMI-based callers outperformed the raw-reads-based callers slightly at each VAF level, while SiNVICT, outLyzer, and Pisces outperformed the UMI-based callers at a VAF of 2.5% or higher in terms of sensitivity.

### Impact of sequencing depth on low-frequency variant callers

**Figure 2:**
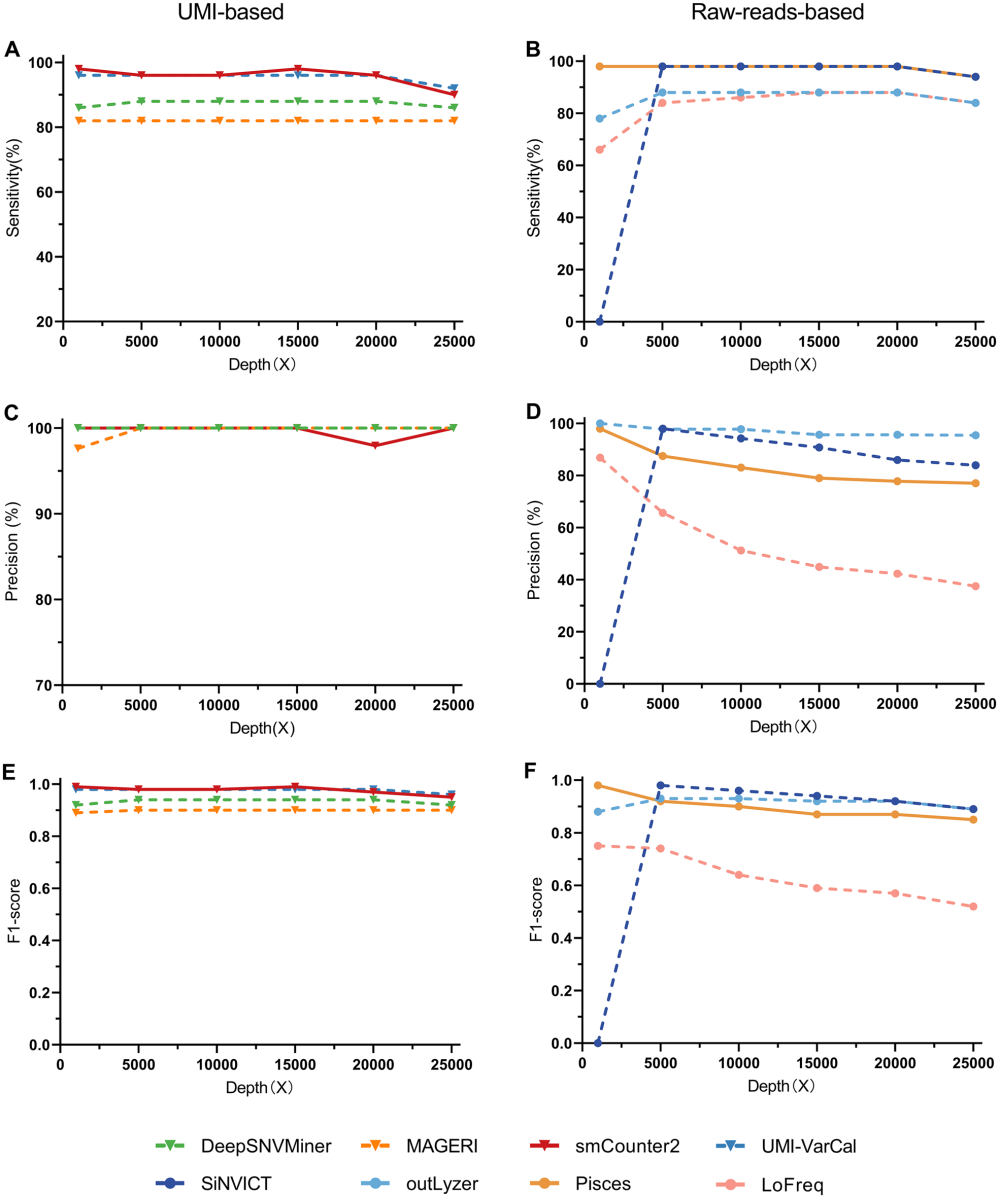
The sensitivity, precision, and F1-score of each low-frequency variant caller concerning sequencing depth. (**A**) The sensitivity of the UMI-based callers**.** (**B**) The sensitivity of the raw-reads-based callers. (**C**) The precision of the UMI-based callers. (**D**) The precision of the raw-reads-based callers. (**E**) The F1-score of the UMI-based callers. (**F**) The F1-score of the raw-reads-based callers. The VAF was 2.5%.

A fundamental solution to improve the accuracy of low-frequency variant calling is to intentionally produce and sequence redundant molecular copies, which requires relatively higher raw sequencing depth than conventional NGS technology^1^. Thus, it is crucial to explore the impact of sequencing depth on low-frequency variant calling tools. The simulated datasets with a range of sequencing depths (1000X, 5000X, 10,000X, 15,000 X, 20,000X, and 25,000X) at a VAF of 2.5% were applied. Figure 2A and B showed the impact of sequencing depth on the sensitivity of each low-frequency variant caller. As shown, the sensitivity of each UMI-based caller remained constant at different depths (Fig. 2A). When the sequencing depth was less than 20,000X, MAGERI and UMI-VarCal remained constant as the sequencing depth increased, with a sensitivity of approximately 82% and 96%, respectively. Simultaneously, DeepSNVMiner's sensitivity tended to rise slightly from 86 to 88% before plateauing at 88%. Except for MAGERI, each low-frequency variant caller significantly decreased when the sequencing depth exceeded 20,000X. As for the raw-reads-based callers in Fig. 2B, When the sequencing depths spanned between 5000 and 20,000X, the sensitivity of each caller remained constant at different depths, although there was a minor decline at 25,000X. When the sequencing depth was less than 5000X, Pisces remained constant, LoFreq and outLyzer tended to increase rapidly, and SiNVICT showed a significant jump from 0 to 98%. Altogether, sequencing depth had no effect on performance for the UMI-based callers when it was less than 20,000X. However, sensitivity and precision decreased when the sequencing depth exceeded 20,000X. Similarly, sequencing depth had almost no effect on the raw-reads-based callers when the sequencing depth was greater than 5000X and less than 20,000X, but the impact became greater in other cases.

In addition, the precision of eight low-frequency variant callers concerning various sequencing depths was shown in Figs. 2C and D. For the UMI-based callers, similar to sensitivity, the sequencing depth had little effect on the precision of the UMI-based callers. DeepSNVMiner and UMI-VarCal had excellent precision of 100% regardless of sequencing depth, while MAGERI and smCounter2 enabled to call variants with high precision (> 97%). For the raw-reads-based callers, the precision of each caller appeared more susceptible at various sequencing depths. For example, as the sequencing depth increased, the precision of outLyzer, LoFreq, and Pisces tended to decrease. Among them, LoFreq and Pisces tended to decrease rapidly at lower sequencing depths (< 15,000X), and then the decrease speed slowed. However, unlike the other three variant callers (outLyzer, LoFreq, and Pisces), the precision of SiNVICT first increased considerably from 0 to 98% and then decreased slowly from a sequencing depth of 5000X to 25,000X.

Regarding the F1-score, a similar case was observed in Figs. 2E and F. The UMI-based variant callers performed very consistently as the sequencing depth increased, while the raw-reads-based variant callers were more susceptible to varying depths. Specifically, SiNVICT and outLyzer showed an increasing trend in the F1-score when the sequencing depth was less than 5000X, while Pisces and LoFreq exhibited a decreasing trend. At a sequencing depth greater than 5000X, SiNVICT and Pisce showed a slight decrease, while outLyzer reached the maximum F1-score of 0.93 at 5000X and 10,000X. In comparison with other callers, LoFreq exhibited a relatively significant decreasing trend.

### Comprehensive analysis for low-frequency variant callers on reference datasets

To comprehensively analyze the eight low-frequency variant callers, reference dataset N0015 with a VAF level of around 5% was first applied (Table 3). To save memory consumption, we only focused on chromosome 1 of N0015, which contained 338 known SNVs. As shown, smCounter2 and Pisces were the only two variant callers capable of detecting all 338 variants, reaching the best performance with 100% sensitivity. Although outLyzer only reported 274 out of 338 variants with a sensitivity of 81.07%, the other variant callers' sensitivity ranged between 96 and 98.5%. Thus, most tools were highly sensitive to N0015 data. Concerning precision, although the raw-reads-based callers (SiNVICT, outLyzer, Pisces, and LoFreq) outperformed the UMI-based callers (DeepSNVMiner, MAGERI, smCounter2, and UMI-VarCal) overall, none of them featured more 67% precision. Among eight variant callers, the precision of SiNVICT, outLyzer, DeepSNVMiner, and smCounter2 ranged between 64.43 and 66.67% while Pisces, LoFreq, and UMI-VarCal featured 57.29%, 54.61%, and 40.12% respectively. The lowest precision of 24.13% was achieved by MAGERI. Finally, the F1-score, the harmonic average of the sensitivity and precision, was then computed. Except for UMI-VarCal and MAGERI, all variant callers performed similarly, with the F1-score ranging from 0.7 to 0.78. In particular, UMI-VarCal had a considerably lower F1-score of 0.57, while MAGERI had the lowest performance, with the F1-score as low as 0.39.

**Table 3:** Performance of eight low-frequency variant callers on the reference datasets N0015 and N13532.

Dataset	Tool	No. of calls ^*a*^	No. of variants ^b^	True positive	False positive	Sensitivity (%)	Precision (%)	F1-score	Accuracy (%)
N0015 (VAF ≈ 5%)	DeepSNVMiner	499	338	328	171	97.04	65.73	0.78	99.37
	MAGERI	1351	338	326	1025	96.45	24.13	0.39	96.37
	smCounter2	516	338	338	178	100	65.5	0.79	99.38
	UMI-VarCal	810	338	325	485	96.15	40.12	0.57	98.26
	SiNVICT	506	338	326	180	96.45	64.43	0.77	99.33
	outLyzer	411	338	274	137	81.07	66.67	0.73	99.30
	Pisces	590	338	338	252	100	57.29	0.73	99.18
	LoFreq	608	338	332	276	98.22	54.61	0.7	99.04
N13532 (VAF ≈ 0.5%)	DeepSNVMiner	31	17	15	16	88.24	48.39	0.62	99.94
	MAGERI	271	17	12	259	70.59	4.43	0.08	99.13
	smCounter2	31	17	17	14	100	54.84	0.71	99.95
	UMI-VarCal	92	17	17	75	100	18.48	0.31	99.75
	SiNVICT	23	17	1	22	5.88	4.35	0.05	99.87
	outLyzer	12	17	1	11	5.88	8.33	0.07	99.91
	Pisces	23	17	1	22	5.88	4.35	0.05	99.97
	LoFreq	752	17	14	738	82.35	1.86	0.04	97.56

The variant allele frequencies of N0015 and N13532 were around 5% and 0.5%, respectively.

^*a*^number of calls, ^*b*^number of variants.

It has been reported that most of the eight low-frequency variant callers had a detection limit below 1% in the original papers (Table 1). Thus, we further explored their performance to low-frequency variant calling using another reference dataset, N13532, with a VAF level of around 0.5%. This dataset contained 17 known SNVs. The results were shown in Table 3. The UMI-based callers discovered substantially more variants than the raw-reads-based callers. Furthermore, except for SiNVICT, outLyzer, and Pisces, all tools retained good sensitivity on dataset N13532. Specifically, UMI-VarCal and smCounter2 reported all 17 variants with a sensitivity of 100%. DeepSNVMiner, LoFreq, and MAGERI followed, reporting 15, 14, and 12 variants, respectively. SiNVICT, outLyzer, and Pisces all detected only one true variant. In terms of precision, smCounter2 scored the highest precision (54.85%), followed by DeepSNVMiner (48.39%) and UMI-VarCal (18.48%). Other tools had a precision of less than 10%. As for the F1-score, only smCounter2 achieved a relatively high F1-score (0.71), followed by DeepSNVMiner (0.62) and UMI-VarCal (0.31), whereas the rest of the callers showed very poor performance (< 0.1). Furthermore, accuracy is also one of the most commonly used metrics. Thus, using reference datasets N0015 and N13532, we calculated the accuracy of each tool. As a result, all the tools achieved accuracy levels above 95%, with some even reaching as high as 99% (Table 3).

### The analysis of substitution signatures

**Figure 3:**
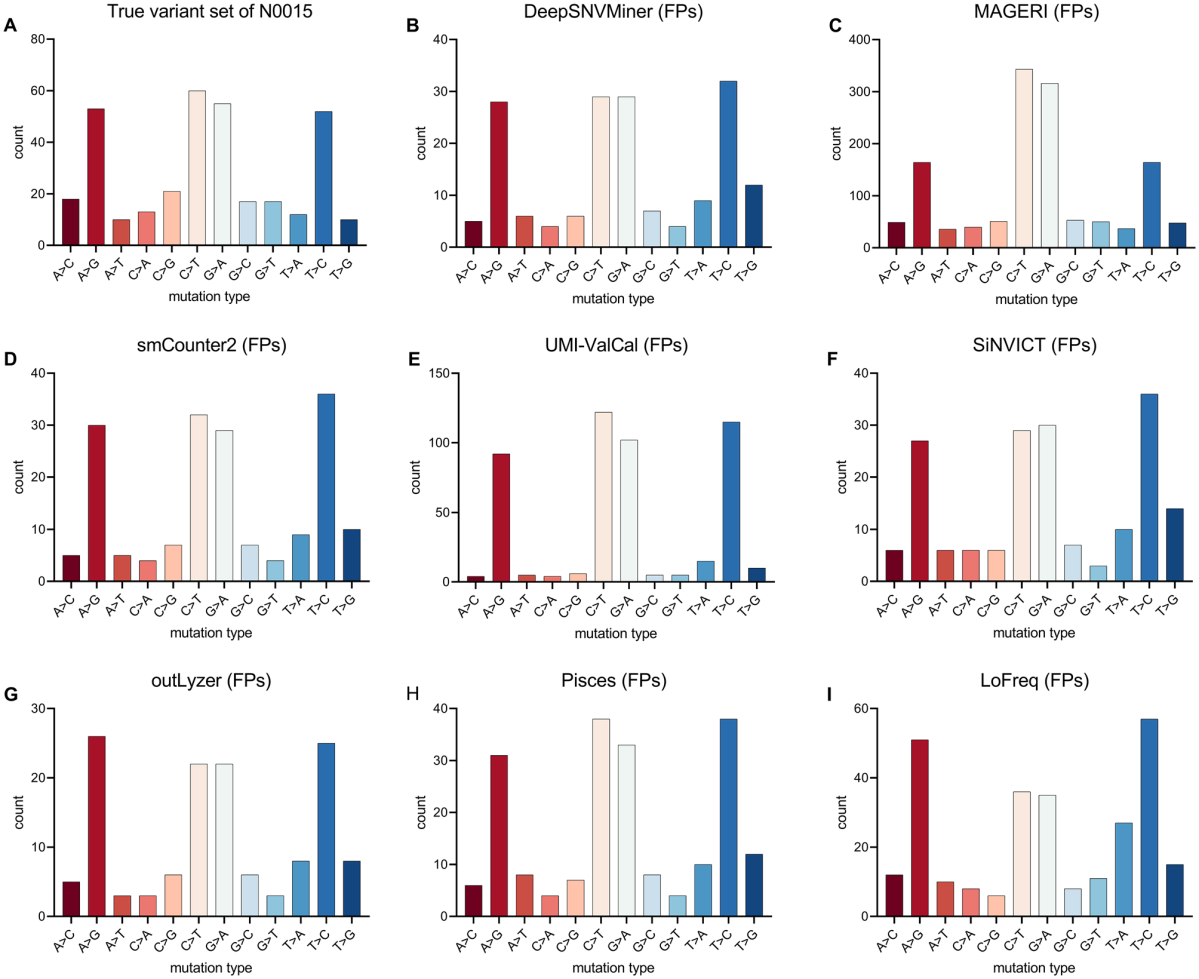
The distribution of substitution signature in the dataset N0015 by using various low-frequency variant callers. (**A**) The distribution of true variant sets in the dataset N0015. (**B–I**) The distribution of substitution signature of false positive variants in the dataset N0015 by using DeepSNVMiner, MAGERI, smCounter2, UMI-VarCal, SiNVICT, outLyzer, Pisces, and LoFreq, respectively.

To examine the substitution signature of false positive variants in the eight low-frequency variant callers, the reference dataset N0015 was analyzed. The substitution signatures and contexts of true and false positive variants obtained using the eight callers were shown in Figs. 3A and B–I, respectively. It demonstrated that there were more calls with T>A and T>G substitutions in the false positive variants called by DeepSNVMiner, smCounter2, SiNVICT, outLyzer, Pisces, and LoFreq than the expected distribution shown in the true variant set of N0015 (Fig. 3A). In comparison, false positives called by MAGERI and UMI-VarCal seem to be independent concerning the base change. However, these false positive variants of MAGERI and UMI-VarCal were unusually high. For MAGERI, all types of base changes were higher than the expected distribution, especially for C>T and G>A. Considering UMI-VarCal, there were more calls with four substitutions A>G, C>T, G>A, and T>C. A certain SNV-type bias in the real data indicated a pipeline-specific feature.

### Application on Horizon Tru-Q sample data

**Figure 4:**
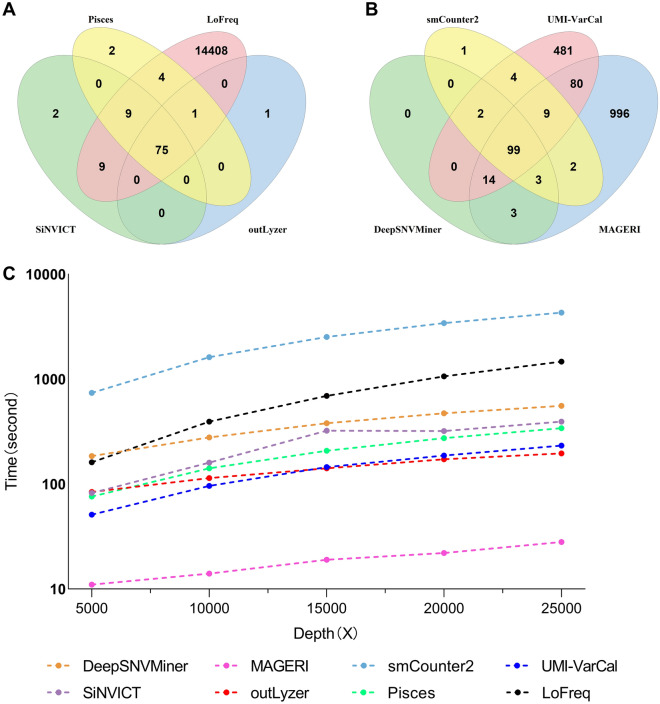
Venn diagrams of the called variants and the execution time of eight low-frequency variant callers. (**A**) Venn diagram showing the number of called variants by the raw-reads-based callers, including SiNVICT, outLyzer, Pisces, and LoFreq. (**B**) Venn diagrams showing the number of called variants by the UMI-based callers, including DeepSNVMiner, MAGERI, smCounter2, and UMI-VarCal. (**C**) Time consumption of eight low-frequency variant callers for variant calling process.

To evaluate the performance of the eight tools on the sample data, Horizon Tru-Q sample data were analyzed, and the variants called by both the raw-reads-based callers (SiNVICT, outLyzer, Pisces, and LoFreq) and the UMI-based callers (DeepSNVMiner, MAGERI, smCounter2, and UMI-VarCal) were illustrated in Figs. 4A and B. For the raw-reads-based callers, a total of 14,511 variants were discovered. Among them, SiNVICT detected 95 variants, whereas outLyzer, Pisces, and LoFreq discovered 77, 91, and 14,506 variants, respectively. Of the 95 variants called by SiNVICT, all four callers shared 75, and 93 were shared by at least one other variant caller. SiNVICT and LoFreq both detected 9 variants whereas Pisces and LoFreq both detected 4 variants. Furthermore, there were 2, 1, 1, and 14,408 distinct variants that SiNVICT, Pisces, outLyzer, and LoFreq, each uniquely called. As for the UMI-based callers, a total of 1694 variants were discovered. DeepSNVMiner, smCounter2, and MAGERI detected 121, 120, and 1206 variants, respectively, while UMI-VarCal detected 689 variants. Among these 121 variants called by DeepSNVMiner, 99 were shared by all four callers, and 119 were shared by at least one other variant caller. Furthermore, the number of variants uniquely called by smCounter2, UMI-VarCal, MAGERI, and DeepSNVMiner was 1, 481, 996, and 3 variants, respectively.

### Comparison of execution time

Execution time is also a vital element for variant callers. Five different samples at various sequencing depths (5000X, 1000X, 15,000X, 20,000X, and 25,000X) were chosen to compare the running time. These samples contain a total of 832,690, 1,669,328, 2,504,424, 3,341,348, and 4,099,748 reads, respectively. All variant calling analyses of different callers were run on a high-performance computing cluster (HPC) of Linux servers at Chongqing University of Posts and Telecommunications. The settings of each node in HPC included 48 Intel(R) Xeon(R) Gold 6136 CPU cores at 3.00 GHz, with a total physical memory of 125 GB and a CentOS Linux release 7 (Core) operating system. The time consumption of each variant caller to complete the analysis was shown in Fig. 4C and Supplementary Table S12. It was obvious that the time to complete the variant calling analysis increased for each tool as the sequencing depth increased. Among the eight low-frequency variant callers, the time spent by LoFreq climbed the fastest when the sequencing depth increased from 5000 to 25,000X, followed by smCounter2, SiNVICT, UMI-VarCal, Pisces, DeepSNVMiner, MAGERI, and the lowest outLyzer. That was, for SiNVICT, UMI-VarCal, Pisces, DeepSNVMiner, and MAGERI, sequencing depth had no significant effect on the running time. In addition, at each sequencing depth, MAGERI was proved to be the fastest running among the eight low-frequency variant callers, with smCounter2 being the slowest. MAGERI, for example, required only ~ 28 s to complete the analysis at a sequencing depth of 25,000X, but smCounter2 required over 1 h. MAGERI outperformed smCounter2 by over 153.64154 times (4302 s vs. 28 s). Therefore, MAGERI was the fastest among the eight variant callers.

## Discussion

Low-frequency variant detection has broad applications in both science and medicine, including cancer, prenatal diagnosis, aging, mutagenesis, antimicrobial resistance, forensics, and so on^1^. However, the detection of significant variants down to 1% has been impeded by the high error rate (0.1–1%) of NGS technologies. To address this issue, a range of low-frequency variant callers have been developed. This study systematically evaluated the performance of eight low-frequency variant callers, including four raw-reads-based variant callers (SiNVICT, outLyzer, Pisces, and LoFreq) and four UMI-based variant callers (DeepSNVMiner, MAGERI, smCounter2, and UMI-VarCal) to detect low-frequency variants by using simulated datasets, reference datasets, and Horizon Tru-Q sample dataset. The results provided crucial theoretical support for the future development of low-frequency variant calling techniques and algorithms. This study is the first to analyze the performance of variant calling tools specifically designed to detect variants with low allelic frequencies, as low as 1% or even lower.

In general, the UMI-based and the raw-reads-based tools apply different principles to call variants. All the UMI-based variant calling tools apply the same approach that tries to correct technical artifacts by performing a majority vote within a UMI family to build a consensus read for each UMI family, and then they apply a statistical method to model background error rate at each position and standard filters to call final variants. In contrast, the raw-reads-based tools utilize a statistical method to model background error rate at each position and apply standard filters to call final variants. There are some similarities and differences in the use of models between these two types of tools (Table 1). For SiNVICT, Pisces, UMI-VarCal, Poisson models are used. However, variant calling procedures are not exactly the same. Poisson model is used in SiNVICT to identify potential variants and to test if the VAF is significantly lower than 0.5. SNV calling in Pisces is determined by a q-score based on reference and non-reference read counts and a Poisson model. UMI-VarCal searches for candidate positions using hypothesis testing. It loops through the pileup and applies a Poisson distribution to calculate the p-value and then applies the Benjamini-Hochberg procedure to obtain the q-value. For smCounter2, LoFreq, and MAGERI, they all use statistical models, but there are some differences. Both smCounter2 and MAGERI require a generating of consensus read for each UMI group of reads, but they take Binomial distribution and Beta-binomial modeling approaches, respectively. Also, LoFreq employs a statistical model that takes into account factors such as base quality scores, mapping quality, and read coverage to identify variants. Given an alignment of reads to a consensus reference, LoFreq treats each base in a column as arising from a Bernoulli trial (success = reference base; failure = variant base). Each trial is assumed to be independent with an associated sequencing error probability that could be derived from a phred-scaled quality value (Q) for the base (P = 10 exp (− Q/10)). This approach leads to a relatively higher number of true positive variants than other methods but is also accompanied by a high number of false positive variants. This could explain why our study has found so many true positive and false positive variants by LoFreq. Additionally, unlike the other tools, DeepSNVMiner applied SAMtools calmd to first generate an initial list of variants and then select the high-confidence variants with strong UMI support.

On the other hand, the UMI-based methods utilize unique molecular identifiers to correct PCR and sequencing errors. These methods aim to reduce false positives and increase the accuracy of variant calling, especially for low-frequency variants. By incorporating UMIs, they can effectively distinguish true variants from sequencing errors and achieve higher precision. However, their precisions on real datasets N0015, N13532, and M0253 were much lower than those on simulated datasets. It might be owing to the reason when analyzing the real datasets, the wrong UMI were devided into the UMI family, resulting in the false positive variants obtainning a high UMI support.

Besides, machine learning (ML) has been deliberated as a core technology in artificial intelligence (AI), and a variety of ML-based variant calling tools has been proposed such as SomaticSeq^38^, SNooPer^39^, NeuSomatic^40^, DeNovoCNN^41^ and so on. SomaticSeq is an ensemble variant caller that requires the union of variant calls from other software as a starting point and then applies its classifiers to remove false positives and uses an adaptive boosting algorithm for classification. However, it is designed for matched tumor-normal variant calling. SNooPer trains a random forest classifier and supports single-sample mode but is designed to work on low-coverage data. NeuSomatic is the first deep-learning-based somatic variant detection framework. NeuSomatic utilizes the effective implementation of convolutional neural networks to solve the problem of somatic variant detection quickly and accurately. DeNovoCNN is a new method based on the convolutional neural network to call variants from de novo mutations (DNMs) and it consistently outperformed other methods in terms of accuracy, recall, and specificity. The utilization of ML for variant calling is totally apt to analyze a large amount of data. While it is still in its infant stages, ML-based variant calling tools hold the promise of updating areas such as clinical genetics.

In general, sensitivity is the probability that a reference variant is called as a variant while precision estimates the probability that a variant call is truly a reference variant. Sensitivity and precision are both crucial metrics for evaluating the performance of these variant calling tools in both clinical and non-clinical life science settings. In clinical settings, sensitivity plays an important role in many areas, such as diagnostics, therapy guidance, prognosis of diseases, and so on^1^. For example, in clinical diagnostics, especially for genetic diseases or cancer, missing a true variant may result in misdiagnosis, inadequate treatment, or failure to identify a patient's susceptibility to certain conditions. In non-clinical settings, both sensitivity and precision remain crucial as well, especially for research and academic purposes. For example, in prenatal genetic testing, precision is crucial to prevent false-positive results, which could lead to unwarranted invasive procedures for pregnant individuals. On the other hand, precision remains important to ensure the quality and validity of research findings.

Our study has comprehensively evaluated the performance of eight low-frequency variant calling tools. According to our analysis of simulated datasets, UMI-VarCal demonstrated the highest sensitivity (> 0.9) for confident low-frequency variant detection at VAFs ranging from 5% to 0.1%. Following closely behind were DeepSNVMiner and MAGERI. smCounter2 was unable to call variants present with VAFs less than 2.5% at any sequencing depth, suggesting a detection limit of 2.5% (Table 2). Several factors could be responsible for smCounter2's increased detection limit when compared to the original paper^17^. First, smCounter2 was a stand-alone variant caller that took binary alignment map (BAM) data as input^17^. However, generating the specific input BAM data without guidelines was difficult since smCounter2 was no longer an open source. Second, a specific error model built for the QIAseq targeted DNA-seq rather than universal protocols was applied. Additionally, at a VAF of 0.025%, none of the variant callers were able to successfully call any variants. When the VAF was more than 5%, the raw-reads-based variant callers outperformed the UMI-based variant callers. However, when VAFs were between 0.05% and 5%, the UMI-based calls were more effective (Table 2). This might be owing to the UMI-based callers’ robust error suppression, which discriminated technical artifacts from errors by performing a majority vote within a UMI family to build a consensus read for each UMI family^10^.

When considering the impact of sequencing depth on the performance of eight variant callers, it was obvious that the sensitivity, precision, and the F1-score of the UMI-based variant callers were less affected by sequencing depth compared to the raw-reads-based variant callers (Fig. 2). The performance of the UMI-based variant callers was nearly consistent across different sequencing depths, while the raw-reads-based variant callers were more affected by varying sequencing depths. In particular, the sensitivity, precision, and F1-score of the UMI-based variant callers showed a slight decrease, except for smCounter2, when the sequencing depth exceeded 20000X. (Fig. 2). This might be due to mechanisms they employ. The UMI-based variant callers are designed to process the short-read sequencing data with unique molecular identifiers. UMIs are incorporated in the library preparation step to trace back to the origin of the DNA molecule. Reads sharing the same UMI are grouped into the same read family. Only families with members above a certain threshold are collapsed to form a consensus sequence, making the UMI-based strategies largely rely on redundant sequencing to obtain enough reads to generate a consensus sequence. Thus, the UMI-based tools were less affected by sequencing depth. However, the raw-reads-based tools rely on the alignment to reference genome to distinguish sequencing errors from actual variants, and this process is highly influenced by the sequencing depth.

Two high-confidence benchmark datasets, N0015 and N13532, were applied to evaluate the performance of these low-frequency variant callers. First, for N0015, confident calling variants could be achieved by all callers with high sensitivity (> 80%). Among them, smCounter2 and Pisces performed the best, with a sensitivity of 100% (Table 3). Concerning precision, none of the variant callers achieved good performance, with all tools ranging from 24.13% to 66.67%. As for the F1-score, all variant calling tools observed a relatively stable F1-score (> 0.70) except for MAGERI and UMI-VarCal, whose F1-scores were 0.39 and 0.57, respectively. For accuracy, all of the tools had accuracy levels above 95%, and some even reached as high as 99% (Table 3). Second, for N13532, smCounter2 and DeepSNVMiner showed good sensitivity and relatively higher precision, whereas MAGERI, LoFreq, and UMI-VarCal featured high sensitivity but low precision. When tested on the dataset N13532 at a VAF of 0.5%, smCounter2 had a sensitivity of 100% and a precision of 54.84%. However, when tested on the simulated datasets at a VAF of 0.5%, smCounter2 had a sensitivity and accuracy of 0% (Supplementary Table S7). The reason that smCounter2 outperformed the simulated datasets on reference datasets was most likely due to its core model being specific to the QIAseq targeted panel sequencing protocol (N0015 and N13532) rather than universal protocols (simulated datasets)^17^. As for the F1-score, all variant calling tools, except for smCounter2, observed the F1-score < 0.70. Therefore, among the eight low-frequency variant calling tools, smCounter2 performed the best, followed by DeepSNVMiner, which also showed relatively good performance. For accuracy, all the tools reached accuracy levels above 95% and some even as high as 99% (Table 3).

In terms of execution time, the eight variant callers performed differently. MAGERI was consistently quick at calling variants. However, smCounter2 took the longest to complete the analysis. As the sequencing depth increased, smCounter2 and LoFreq consumed significantly more time than the others (Supplemental Table S12).

The validation of low-frequency variant calling revealed that the UMI-based variant calling tools outperformed the raw-reads-based variant calling tools in terms of detection limit. Compared to the original papers^9,15,16^, the UMI-based variant calling tools, like DeepSNVMiner, MAGERI, and UMI-VarCal, were able to lower their detection limit to 0.05%. However, smCounter2 did not show similar improvement due to its error model being specific to the QIAseq targeted panel sequencing protocol rather than universal sequencing protocols. Thus, smCounter2 was not recommended. Additionally, previous studies have shown that SiNVICT and outLyzer were capable of detecting SNVs with VAF as low as 0.5% and 1%, respectively^5,6^. However, confident calling could be only achieved with VAF > 2.5% based on analysis of both real datasets and simulated datasets, which showed lower sensitivity than in previous studies. Similarly, LoFreq, which had a lower sensitivity of 0.25% in earlier research^8^, reliably called variants with VAFs > 0.5%. Even though LoFreq was able to attain better sensitivity, there was always a trade-off between precision and sensitivity.

## Conclusions

In conclusion, a total of eight low-frequency variant calling tools, including four raw-read-based tools and four UMI-based tools, were comprehensively evaluated in this study. Our benchmarking on simulated and real datasets revealed that the UMI-based variant callers, except smCounter2, outperformed the raw-reads-based variant callers regarding detection limit. The performance of the UMI-based callers was nearly constant, regardless of the sequencing depths. Additionally, MAGERI could perform the quickest analysis regardless of sequencing depth, whereas smCounter2 always took the longest time to complete variant calling. For calling variants present with ultra-low frequency, we recommended UMI-VarCal and DeepSNVMiner since they delivered the best results in terms of sensitivity and precision. Also, smCounter2 was a suitable option when the user was enabled to figure out how to generate its specific input BAM file. Our results facilitate the benchmarking analysis of reliable low-frequency variant detection, which is critical in genetics-based medical research and clinical applications. We hope that our results provide important information regarding future directions for the development of low-frequency calling algorithms.

## Methods

### Sources of benchmarking dataset acquisition

#### Simulated datasets

As an open-source simulator for targeted sequencing paired-end data, UMI-Gen (https://gitlab.com/vincent-sater/umigen) can generate the UMI-based reference reads using a number of control files to estimate the background error rate at each position and then modify the generated reads to mimic real short-read data^35^. UMI-Gen requires a minimum of three parameters at execution: a list of control BAM/SAM samples, the BED file with the coordinates of the targeted genomic regions, and a reference genome FASTA file with a BWA index file. Three control samples in BAM format obtained from test data in the original paper were used in this study to estimate the background error rate of the sequencing platform. For the required BED file, a list of regions was selected from test data in the original paper to define the target sequencing panel (Table S1). The version hg19 of the human reference genome (i.e. GRCh37) was downloaded from UCSC (https://hgdownload.soe.ucsc.edu/goldenPath/hg19/bigZips/latest/hg19.fa.gz). In addition, a CSV file containing a list of variants with known variant allele frequency (VAF) was provided (Table S2). In this study, 50 SNVs were randomly inserted into the simulated reads, and the VAFs of the known SNVs were set to 9 different levels (10%, 5%, 2.5%, 1%, 0.5%, 0.25%, 0.1%, 0.05%, and 0.025%) to evaluate the detection limit. Also, at each VAF level, a series of sequencing depths (1000X, 5000X, 10,000X, 15,000X, 20,000X, and 25,000X) were produced to investigate the impact of sequencing depths on the low-frequency variant callers. Therefore, a total of 54 data points (6 × 9 = 96) with UMI-tagged paired-end reads (2 × 110 bp), varying VAF levels, and read depths were generated. For each read pair, they shared the same UMI tag that was linked to the end of the read name by “_” (i.e. @XXXX_NNNNNNNNNNNN).

#### Reference datasets

For better evaluation of the performance of each low-frequency variant caller, two reference datasets N0015 and N13532 from Chang Xu et al. were applied^17^. Both N0015 and N13532 originated from a mixture of two different proportions of NA12878 DNA and NA24385 DNA to simulate low-frequency variants. N0015 was generated by sequencing a mixture of 10% NA12878 DNA and 90% NA24385 DNA, while N13532 was generated by sequencing a mixture of 1% NA12878 DNA and 99% NA24385 DNA, respectively. N0015 and N13532 contained high-confidence variants released by GIAB (v3.3.2 used for Chang Xu et al.^17^) with VAF around 5% and 0.5%, respectively. In both two datasets, read 1 started with the primer, while read 2 started with 8 bp UMI tags, followed by 11 bp common sequence 5‘-ATTGGAGTCCT-3’. Sequencing was performed using the Illumina NextSeq 500 machine. In this study, we only focused on regions on chromosome 1 of N0015 and chromosome 10 of N13532 to save time consumption, which contained 338 SNVs and 17 SNVs, respectively.

#### Horizon Tru-Q sample data

Horizon Tru-Q sample M0253 was downloaded from Chang Xu et al.^17^. It was generated by sequencing the Tru-Q 7 reference standard (Horizon Dx) that contained verified 1.0% (and above) onto specific variants. The sample was diluted 1:1 in Tru-Q 0 (wild-type, Horizon Dx) to simulate 0.5% variants. For the M0253, we only had a list of variants that Tru-Q 7 contained.

### Data pre-processing

Prior to the variant calling, raw sequencing reads in FASTQ files needed to be pre-processed to filter out low-quality reads. The flowchart of benchmarking analysis of different variant calling tools was illustrated in Supplementary Fig. S1. Quality control and filtering of raw sequencing reads were first performed using FASTP v0.23.2. The high-quality reads were then aligned with the human reference genome GRCh37 using BWA-MEM v0.7.17-r1188 to generate the alignment file in BAM format. Afterward, SAMtools v1.9 was used to perform post-alignment filtering to remove secondary (256-bit), supplementary (2048-bit), read unmapped (4-bit), and mate unmapped (8-bit) alignments while retaining reads mapped in proper pair (2-bit) alignments. Finally, the Viterbi module of LoFreq v2.1.5 was applied to conduct base quality score recalibration (BQSR) to improve the mapping accuracy and reduce the noise introduced by the mappings.

### Variant calling

The simulated datasets were divided into 6 groups according to the read depth (1000X, 5000X, 10,000X, 15,000X, 20,000X, and 25,000X), and each group contained 9 different levels of VAFs (10%, 5%, 2.5%, 1%, 0.5%, 0.25%, 0.1%, 0.05%, and 0.025%). Thus, a total of 54 simulated datasets (6 × 9 = 54) were generated and applied. A custom-made BED file was created by using BEDtools v2.29.1 to call variants only in interested regions. The eight variant callers including four raw-reads-based variant callers (SiNVICT, outLyzer, Pisces, and LoFreq) and four UMI-based variant callers (DeepSNVMiner, MAGERI, smCounter2, and UMI-VarCal) were then performed in parallel using simulated data, reference data, and Horizon Tru-Q sample data, respectively. Summaries of each low-frequency variant caller were shown in Table 1. In addition, all command lines used in this work were shown in Supplementary Methods S1. To conduct a fair comparison of eight tools, we stick to the default recommended options except for DeepSNVMiner, smCounter2, and UMI-VarCal with a few tweaks. Finally, a list of original VCF format files was generated by these tools. Note that indels detected from sequencing data would be discarded since we only focused on the performance of eight tools for SNVs calling.

### Performance evaluation metrics

For an assessment of each variant caller’s performance, an in-house Python script was used to enumerate the variants between the true variant sets and the called sets which contained the output variants of the variant callers. Each variant caller’s performance was measured statistically as sensitivity, precision, accuracy, and F1-score, as shown in Eqs. (1), (2), (3), and (4), respectively. Especially, as the harmonic mean of sensitivity and precision, the F1-score was used as the key metric for evaluating the performance of the pipelines.

1 $$\begin{array}{c}Sensitivity=\frac{TP}{TP+FN}\end{array}$$

2 $$\begin{array}{c}Precision=\frac{TP}{TP+FP}\end{array}$$

3 $$\begin{array}{c}Accuracy=\frac{TP+TN}{TP+TN+FP+FN}\end{array}$$

4 $$\begin{array}{c}F1-score=\frac{2\times sensitivity\times precision}{sensitivity+precision}\end{array}$$

where TP (true positive) was a variant that existed in the true variant sets and was also detected by the callers; TN (true negative) was a variant that did not exist in the true variant sets and was also not detected by the callers. FP (false positive) was a variant that did not exist in the true variant sets but was detected by the callers; FN (false negative) was a variant that existed in the true variant sets but was not detected by the callers.

### Supplementary Information

Supplementary Information.

## Data Availability

The data that support the findings of this study are available from Sequence Read Archive (SRA). N0015, N13532 and M0253 are under accession number SRR3493407, SRX4395160 and SRX4395159, respectively. The simulated data used in this study can be generated and a detailed tutorial is openly available at GitLab (https://gitlab.com/vincent-sater/umigen).
