# Supplementary material for: Evaluating the performance of low-frequency variant calling tools for the detection of variants from short-read deep sequencing data
Source: Sci Rep. 2023 Nov 22;13:20444. doi: 10.1038/s41598-023-47135-3 (PMC10665316; doi:10.1038/s41598-023-47135-3)
Supplement: Supplementary file 1 — Supplementary Information. [file 41598_2023_47135_MOESM1_ESM.pdf]

Supplementary Information
for
Evaluating the performance of low-frequency variant calling tools for the detection of variants from short-read deep sequencing data

Xudong Xiang [a], Bowen Lu [a], Dongyang Song [a], Jie Li [a], Kunxian Shu [a, *], Dan Pu [a, *]

[a] Chongqing Key Laboratory of Big Data for Bio Intelligence, Chongqing University of Posts and Telecommunications, Chongqing, China, 400065.

* To whom correspondence should be addressed. E-mail: shukx@cqupt.edu.cn; pudan@cqupt.edu.cn

Supplementary Methods

Generation of simulated data

We used the following UMI-Gen command for the generation of simulated datasets. To generate data containing mutations with various VAFs at different sequencing depths, we set **-d** to 1000, 5000, 10000, 15000, 20000, and 25000, and variant allele frequency in **variants.csv** was set to 0.1, 0.05, 0.025, 0.01, 0.005, 0.0025, 0.001, 0.0005, 0.00025, respectively. Therefore, a total of 54 sets of UMI-tagged paired-end reads (2×110bp) with various VAF levels and sequencing depths were generated.

Python3 umi-gen.py snv -i Control_1.bam, Control_2.bam, Control_3.bam -f Ref_hg19.fa -b Target_region.bed **-v Variants.csv** -o Output_dir -n Name **-d Depth**

Data processing pipelines

1. Simulated data

For input data of SiNVICT, outLyzer, Pisces, LoFreq, UMI-VarCal, and smCounter2, we conducted the following processing pipeline. Note that the last additional step was performed only for input data of smCounter2.

a. Read filtering (FASTP 0.23.2)

   fastp -A -i Raw_R1.fastq -I Raw_R2.fastq -o QCed_R1.fastq -O QCed_R1.fastq

b. Read alignment (BWA-MEN 0.7.17-r1188)

   bwa mem -t 36 Ref_hg19.fa QCed_R1.fastq QCed_R1.fastq >input.sam

c. Post alignment filtering (SAMtools 1.9)

   i. samtools view -Sb -F 2316 -f 2 -@ 36 input.sam | samtools sort -@ 36 -o input_Ff.bam

   ii. samtools index -@ 36 input_Ff.bam

d. Base quality score recalibration (LoFreq 2.1.5)

   i. LoFreq viterbi -f Ref_hg19.fa -k input_Ff.bam | samtools sort -@ 36 -o input_recal.bam

   ii. samtools index -@ 36 input_recal.bam

e. Add UMI to Mi tag (**Note: for smCounter2 only**)

   Python3 add_UMI.py input_recal.bam input_recal_Mi.bam

For input data of DeepSNVMiner and MAGERI, we conducted the following processing pipeline.

a. Read filtering (FASTP 0.23.2)

   fastp -A -i Raw_R1.fastq -I Raw_R2.fastq -o QCed_R1.fastq -O QCed_R1.fastq

b. Extraction of UMI tags

- Python3 extract_umi_MAGERI.py QCed_R1.fastq QCed_R1.fastq MAGERI_R1.fastq MAGERI_R2.fastq (For MAGERI)
- Python3 extract_umi_DeepSNVMiner.py QCed_R1.fastq QCed_R1.fastq DeepSNVMiner_R1.fastq DeepSNVMiner_R2.fastq (For DeepSNVMiner)

2. Reference data and Horizon Tru-Q sample data

For input data of SiNVICT, outLyzer, Pisces, LoFreq, and UMI-VarCal, we conducted the following processing pipeline. Note that the last step was performed for input data of smCounter2.

a. Read filtering (FASTP 0.23.2)

   fastp -A -i Raw_R1.fastq -I Raw_R2.fastq -o QCed_R1.fastq -O QCed_R1.fastq

b. Read trimming

   Python3 read_trimmer.py –-r1 QCed_R1.fastq –-r2 QCed_R2.fastq –-out-r1 Trimmed_R1.fastq –-out-r2 Trimmed_R2.fastq –-out-metrics Metrics.txt --primer-file Primer3.txt --primer-col 3 –umi-len 12 –common-seq-len 11

c. Read alignment (BWA-MEN 0.7.17-r1188)

   bwa mem -t 36 Ref_hg19.fa Trimmed_R1.fastq Trimmed_R2.fastq >input.sam

d. Post alignment filtering (SAMtools 1.9)

   i. samtools view -Sb -F 2316 -f 2 -@ 36 input.sam | samtools sort -@ 36 -o input_Ff.bam

   ii. samtools index -@ 36 input_Ff.bam

e. Base quality score recalibration (LoFreq 2.1.5)

   i. LoFreq viterbi -f Ref_hg19.fa -k input_Ff.bam | samtools sort -@ 36 -o input_recal.bam

   ii. samtools index -@ 36 input_recal.bam

f. Add UMI to Mi tag (**Note: for smCounter2 only**)

   Python3 add_UMI.py input_recal.bam input_recal_Mi.bam

For input data of DeepSNVMiner and MAGERI, we conducted the following processing pipeline.

a. Read filtering (FASTP 0.23.2)

   fastp -A -i Raw_R1.fastq -I Raw_R2.fastq -o QCed_R1.fastq -O QCed_R1.fastq

b. Read trimming

   Python3 read_trimmer.py –-r1 QCed_R1.fastq –-r2 QCed_R2.fastq –-out-r1 Trimmed_R1.fastq –-out-r2 Trimmed_R2.fastq –-out-metrics Metrics.txt --primer-file Primer3.txt --primer-col 3 –umi-len 12 –common-seq-len 11

c. Extraction of UMI tags

- Python3 extract_umi_MAGERI.py Trimmed_R1.fastq Trimmed_R2.fastq MAGERI_R1.fastq MAGERI_R2.fastq (For MAGERI)
- Python3 extract_umi_DeepSNVMiner.py Trimmed_R1.fastq Trimmed_R2.fastq DeepSNVMiner_R1.fastq DeepSNVMiner_R2.fastq (For DeepSNVMiner)

Variant calling

We used the following SiNVICT command for all datasets.

1. bam-readcount -w1 -f Ref_hg19.fa input_recal.bam -l Region.txt >readcount_dir/ sim_recal.readcount
2. sinvict -t readcount_dir/ -o output_dir/

We used the following outLyzer command for all datasets.

python3 outLyzer.py calling -ref Ref_hg19.fa -bam input_recal.bam -bed Target_region.bed -output output_dir/

We used the following Pisces command for all datasets.

1. CreateGenomeSizeFile -s "Human (UCSC hg19)" -g Ref_hg19_dir/ -o Ref_hg19_dir/
2. Pisces -b input_recal.bam -g Ref_hg19_dir -i Target_region.bed -o output_dir --gvcf false --callmnvs false --filterduplicates true --ssfilter true

We used the following LoFreq command for simulated datasets. For N0015, N13532, M0253, there was no -s set.

LoFreq call-parallel -s -f Ref_hg19.fa -o output.vcf -l Target_region.bed input_recal.bam

We used the following DeepSNVMiner command for simulated datasets. For N0015, N13532, M0253, -sm_count was set to 20 and -min_group was set to 5.

run_deepseq.pl -read1_fastq DeepSNVMiner_R1.fastq -read2_fastq DeepSNVMiner_R2.fastq -ref_fasta Ref_hg19.fa -filename_stub output -coord_bed Target_region.bed -working_dir output_dir -no_adaptor -uid_done -sm_count 10 -min_group 2 -bwa /path/to/bwa -samtools /path/to/samtools

We used the following MAGERI command for all datasets.

1. bedtools getfasta -ref Ref_hg19.fa -bed Target_region.bed --fo Target_region.fa
2. java -Xmx200g -jar MAGERI.jar -M4 -R1 MAGERI_R1.fastq -R2 MAGERI_R2.fastq --bed Target_region.bed --references Target_region.fa -O output_dir --project-name project_name --sample-name sample_name

We used the following smCounter2 command for simulated datasets. For N0015, N13532, M0253, --minAltUMI was

set to 3.

Python2 smCounter2.py --bamFile input_recal.bam --refGenome Ref_hg19.fa --bedTarget Target_region.bed --runPath output_dir --outPrefix output_prefix --minAltUMI 1

We used the following UMI-VarCal command for simulated datasets. For N0015, N13532, M0253, --min_variant_umi was set to 5 and --min_variant_depth was set to 5.

Python3 UMI-VarCal.py call -i input_recal.bam -b Target_region.bed -f Ref_hg19.fa -o output_dir --gvcf False --keep_pileup False --min_variant_umi 1 --min_variant_depth 1

Supplementary Tables

Table S1. List of regions for target sequencing of simulated datasets in the required BED file.

Chromosome	ChromStart	ChromEnd	Chromosome	ChromStart	ChromEnd
chr1	2488028	2488202	chr1	27100803	27101749
chr1	2489134	2489299	chr1	27102061	27102220
chr1	2489769	2489922	chr1	27105487	27107269
chr1	2491197	2491487	chr1	117057340	117057467
chr1	2492024	2492195	chr1	117061767	117061983
chr1	2493106	2493327	chr1	117064459	117064637
chr1	2494277	2494400	chr1	117078575	117078867
chr1	2494550	2494737	chr1	117086892	117087259
chr1	23885399	23885521	chr1	117113455	117113657
chr1	23885571	23885958	chr1	120457915	120459331
chr1	27022885	27024142	chr1	120464845	120465089
chr1	27056127	27056382	chr1	120465250	120465407
chr1	27057601	27058143	chr1	120466253	120466632
chr1	27059133	27059288	chr12	57493709	57493956
chr1	27087326	27087634	chr12	57496008	57496313
chr1	27087766	27087982	chr12	57496569	57496803
chr1	27088634	27088816	chr12	57498218	57498386
chr1	27089442	27089799	chr12	57498477	57498616
chr1	27092687	27092862	chr12	57498919	57499133
chr1	27092942	27093076	chr13	41133651	41135056
chr1	27094275	27094498	chr13	41239711	41240436
chr1	27097600	27097823	chr15	45003694	45003859
chr1	27098985	27099130	chr15	45007612	45007927
chr1	27099295	27099483	chr15	45008466	45008575
chr1	27099831	27100413	chrX	100610986	100611268

Table S2. List of regions for target sequencing of simulated data. 50 known SNVs were randomly generated for the simulation of mutations.

chromosome	position	reference	alternation	chromosome	position	reference	alternation
chr1	2488090	T	G	chr1	27101496	G	T
chr1	2489135	A	T	chr1	27102063	T	A
chr1	2489816	G	A	chr1	27105652	A	G
chr1	2491206	G	C	chr1	117057456	A	C
chr1	2492094	C	G	chr1	117061775	T	C
chr1	2493152	A	G	chr1	117064480	A	C
chr1	2494365	C	G	chr1	117078714	T	G
chr1	2494674	A	G	chr1	117087055	A	C
chr1	23885437	A	T	chr1	117113503	C	T
chr1	23885584	G	T	chr1	120459167	G	T
chr1	27022889	G	A	chr1	120464891	A	C
chr1	27056361	G	A	chr1	120465269	C	G
chr1	27057804	A	T	chr1	120466280	T	C
chr1	27059271	C	T	chr12	57493827	C	A
chr1	27087532	T	G	chr12	57496213	T	C
chr1	27087898	C	G	chr12	57496582	T	A
chr1	27088797	G	A	chr12	57498308	C	G
chr1	27089666	T	G	chr12	57498494	C	A
chr1	27092771	G	T	chr12	57498951	C	T
chr1	27092998	C	G	chr13	41133867	A	C
chr1	27094468	A	T	chr13	41240184	C	A
chr1	27097724	T	G	chr15	45003799	G	A
chr1	27099071	C	A	chr15	45007629	A	G
chr1	27099481	G	A	chr15	45008544	G	C
chr1	27100182	G	T	chrX	100611134	T	G

Table S3. Performance of eight low-frequency variant callers on simulated datasets with different sequencing depths (1000X, 5000X, 10000X, 15000X, 20000X, and 25000X) at a VAF of 10%.

Variant caller	1000X					5000X					10000X				
	NC	TP	FP	S(%)	P(%)	NC	TP	FP	S(%)	P(%)	NC	TP	FP	S(%)	P(%)
DeepSNVMiner	44	44	0	88.00	100.00	44	44	0	88.00	100.00	44	44	0	88.00	100.00
MAGERI	43	41	2	82.00	95.35	41	41	0	82.00	100.00	41	41	0	82.00	100.00
smCounter2	49	49	0	98.00	100.00	49	49	0	98.00	100.00	49	49	0	98.00	100.00
UMI-VarCal	48	48	0	96.00	100.00	48	48	0	96.00	100.00	48	48	0	96.00	100.00
SiNVICT	49	49	0	98.00	100.00	50	49	1	98.00	98.00	53	49	4	98.00	92.45
outLyzer	50	50	0	100.00	100.00	51	50	1	100.00	98.04	51	50	1	100.00	98.04
Pisces	50	49	1	98.00	98.00	57	49	8	98.00	85.96	59	49	10	98.00	83.05
LoFreq	53	48	5	96.00	90.57	63	48	15	96.00	76.19	75	48	27	96.00	64.00

Variant caller	15000X					20000X					25000X				
	NC	TP	FP	S(%)	P(%)	NC	TP	FP	S(%)	P(%)	NC	TP	FP	S(%)	P(%)
DeepSNVMiner	44	44	0	88.00	100.00	44	44	0	88.00	100.00	44	44	0	88.00	100.00
MAGERI	41	41	0	82.00	100.00	41	41	0	82.00	100.00	41	41	0	82.00	100.00
smCounter2	49	49	0	98.00	100.00	49	49	0	98.00	100.00	47	47	0	94.00	100.00
UMI-VarCal	48	48	0	96.00	100.00	48	48	0	96.00	100.00	46	46	0	92.00	100.00
SiNVICT	54	49	5	98.00	90.74	56	49	7	98.00	87.50	56	47	9	94.00	83.93
outLyzer	52	50	2	100.00	96.15	52	50	2	100.00	96.15	50	48	2	96.00	96.00
Pisces	62	49	13	98.00	79.03	62	49	13	98.00	79.03	60	47	13	94.00	78.33
LoFreq	74	48	26	96.00	64.86	84	48	36	96.00	57.14	84	46	38	92.00	54.76

NC: number of calls; NM: number of mutations; TP: true positive; FP: false positive; S: sensitivity; P: precision.

Table S4. Performance of eight low-frequency variant callers on simulated datasets with different sequencing depths (1000X, 5000X, 10000X, 15000X, 20000X, and 25000X) at a VAF of 5%.

Variant caller	1000X					5000X					10000X				
	NC	TP	FP	S(%)	P(%)	NC	TP	FP	S(%)	P(%)	NC	TP	FP	S(%)	P(%)
DeepSNVMiner	44	44	0	88.00	100.00	44	44	0	88.00	100.00	44	44	0	88.00	100.00
MAGERI	43	41	2	82.00	95.35	41	41	0	82.00	100.00	41	41	0	82.00	100.00
smCounter2	49	49	0	98.00	100.00	49	49	0	98.00	100.00	49	49	0	98.00	100.00
UMI-VarCal	48	48	0	96.00	100.00	48	48	0	96.00	100.00	48	48	0	96.00	100.00
SiNVICT	48	48	0	96.00	100.00	50	49	1	98.00	98.00	52	49	3	98.00	94.23
outLyzer	50	50	0	100.00	100.00	51	50	1	100.00	98.04	51	50	1	100.00	98.04
Pisces	50	49	1	98.00	98.00	58	49	9	98.00	84.48	57	49	8	98.00	85.96
LoFreq	50	46	4	92.00	92.00	60	47	13	94.00	78.33	76	47	29	94.00	61.84
**Variant caller**	**15000X**					**20000X**					**25000X**				
	**NC**	**TP**	**FP**	**S(%)**	**P(%)**	**NC**	**TP**	**FP**	**S(%)**	**P(%)**	**NC**	**TP**	**FP**	**S(%)**	**P(%)**
DeepSNVMiner	44	44	0	88.00	100.00	44	44	0	88.00	100.00	44	44	0	88.00	100.00
MAGERI	41	41	0	82.00	100.00	41	41	0	82.00	100.00	41	41	0	82.00	100.00
smCounter2	49	49	0	98.00	100.00	50	49	1	98.00	98.00	47	47	0	94.00	100.00
UMI-VarCal	48	48	0	96.00	100.00	48	48	0	96.00	100.00	46	46	0	92.00	100.00
SiNVICT	54	49	5	98.00	90.74	56	49	7	98.00	87.50	56	47	9	94.00	83.93
outLyzer	52	50	2	100.00	96.15	52	50	2	100.00	96.15	50	48	2	96.00	96.00
Pisces	61	49	12	98.00	80.33	63	49	14	98.00	77.78	61	47	14	94.00	77.05
LoFreq	79	48	31	96.00	60.76	91	48	43	96.00	52.75	91	46	45	92.00	50.55

NC: number of calls; NM: number of mutations; TP: true positive; FP: false positive; S: sensitivity; P: precision.

Table S5. Performance of eight low-frequency variant callers on simulated datasets with different sequencing depths (1000X, 5000X, 10000X, 15000X, 20000X, and 25000X) at a VAF of 2.5%.

Variant caller	1000X					5000X					10000X				
	NC	TP	FP	S(%)	P(%)	NC	TP	FP	S(%)	P(%)	NC	TP	FP	S(%)	P(%)
DeepSNVMiner	44	44	0	88.00	100.00	44	44	0	88.00	100.00	44	44	0	88.00	100.00
MAGERI	43	41	2	82.00	95.35	41	41	0	82.00	100.00	41	41	0	82.00	100.00
smCounter2	49	49	0	98.00	100.00	49	49	0	98.00	100.00	49	49	0	98.00	100.00
UMI-VarCal	48	48	0	96.00	100.00	48	48	0	96.00	100.00	48	48	0	96.00	100.00
SiNVICT	48	48	0	96.00	100.00	50	49	1	98.00	98.00	52	49	3	98.00	94.23
outLyzer	50	50	0	100.00	100.00	51	50	1	100.00	98.04	51	50	1	100.00	98.04
Pisces	50	49	1	98.00	98.00	58	49	9	98.00	84.48	57	49	8	98.00	85.96
LoFreq	50	46	4	92.00	92.00	60	47	13	94.00	78.33	76	47	29	94.00	61.84
**Variant caller**	**15000X**					**20000X**					**25000X**				
	**NC**	**TP**	**FP**	**S(%)**	**P(%)**	**NC**	**TP**	**FP**	**S(%)**	**P(%)**	**NC**	**TP**	**FP**	**S(%)**	**P(%)**
DeepSNVMiner	44	44	0	88.00	100.00	44	44	0	88.00	100.00	44	44	0	88.00	100.00
MAGERI	41	41	0	82.00	100.00	41	41	0	82.00	100.00	41	41	0	82.00	100.00
smCounter2	49	49	0	98.00	100.00	50	49	1	98.00	98.00	47	47	0	94.00	100.00
UMI-VarCal	48	48	0	96.00	100.00	48	48	0	96.00	100.00	46	46	0	92.00	100.00
SiNVICT	54	49	5	98.00	90.74	56	49	7	98.00	87.50	56	47	9	94.00	83.93
outLyzer	52	50	2	100.00	96.15	52	50	2	100.00	96.15	50	48	2	96.00	96.00
Pisces	61	49	12	98.00	80.33	63	49	14	98.00	77.78	61	47	14	94.00	77.05
LoFreq	79	48	31	96.00	60.76	91	48	43	96.00	52.75	91	46	45	92.00	50.55

NC: number of calls; NM: number of mutations; TP: true positive; FP: false positive; S: sensitivity; P: precision.

Table S6. Performance of eight low-frequency variant callers on simulated datasets with different sequencing depths (1000X, 5000X, 10000X, 15000X, 20000X, and 25000X) at a VAF of 1%.

Variant caller	1000X					5000X					10000X				
	NC	TP	FP	S(%)	P(%)	NC	TP	FP	S(%)	P(%)	NC	TP	FP	S(%)	P(%)
DeepSNVMiner	44	44	0	88.00	100.00	44	44	0	88.00	100.00	44	44	0	88.00	100.00
MAGERI	40	39	1	78.00	97.50	41	41	0	82.00	100.00	41	41	0	82.00	100.00
smCounter2	0	0	0	0.00	0.00	0	0	0	0.00	0.00	0	0	0	0.00	0.00
UMI-VarCal	50	48	2	96.00	96.00	48	48	0	96.00	100.00	48	48	0	96.00	100.00
SiNVICT	0	0	0	0.00	0.00	1	0	1	0.00	0.00	5	1	4	2.00	20.00
outLyzer	0	0	0	0.00	0.00	1	0	1	0.00	0.00	2	1	1	2.00	50.00
Pisces	1	0	1	0.00	0.00	9	1	8	2.00	11.11	14	4	10	8.00	28.57
LoFreq	10	7	3	14.00	70.00	45	18	27	36.00	40.00	60	20	40	40.00	33.33
**Variant caller**	**15000X**					**20000X**					**25000X**				
	NC	TP	FP	S(%)	P(%)	NC	TP	FP	S(%)	P(%)	NC	TP	FP	S(%)	P(%)
DeepSNVMiner	43	43	0	86.00	100.00	44	44	0	88.00	100.00	43	43	0	86.00	100.00
MAGERI	41	41	0	82.00	100.00	41	41	0	82.00	100.00	41	41	0	82.00	100.00
smCounter2	0	0	0	0.00	0.00	0	0	0	0.00	0.00	1	0	1	0.00	0.00
UMI-VarCal	48	48	0	96.00	100.00	48	48	0	96.00	100.00	46	46	0	92.00	100.00
SiNVICT	6	1	5	2.00	16.67	8	1	7	2.00	12.50	10	1	9	2.00	10.00
outLyzer	3	1	2	2.00	33.33	3	1	2	2.00	33.33	3	1	2	2.00	33.33
Pisces	18	5	13	10.00	27.78	21	7	14	14.00	33.33	21	7	14	14.00	33.33
LoFreq	81	20	61	40.00	24.69	88	20	68	40.00	22.73	95	22	73	44.00	23.16

NC: number of calls; NM: number of mutations; TP: true positive; FP: false positive; S: sensitivity; P: precision.

Table S7. Performance of eight low-frequency variant callers on simulated datasets with different sequencing depths (1000X, 5000X, 10000X, 15000X, 20000X, and 25000X) at a VAF of 0.5%.

Variant caller	1000X					5000X					10000X				
	NC	TP	FP	S(%)	P(%)	NC	TP	FP	S(%)	P(%)	NC	TP	FP	S(%)	P(%)
DeepSNVMiner	0	0	0	0.00	0.00	44	44	0	88.00	100.00	43	43	0	86.00	100.00
MAGERI	1	0	1	0.00	0.00	41	41	0	82.00	100.00	41	41	0	82.00	100.00
smCounter2	0	0	0	0.00	0.00	0	0	0	0.00	0.00	0	0	0	0.00	0.00
UMI-VarCal	0	0	0	0.00	0.00	48	48	0	96.00	100.00	48	48	0	96.00	100.00
SiNVICT	0	0	0	0.00	0.00	1	0	1	0.00	0.00	3	0	3	0.00	0.00
outLyzer	0	0	0	0.00	0.00	1	0	1	0.00	0.00	1	0	1	0.00	0.00
Pisces	1	0	1	0.00	0.00	9	0	9	0.00	0.00	10	0	10	0.00	0.00
LoFreq	3	0	3	0.00	0.00	28	5	23	10.00	17.86	56	8	48	16.00	14.29
**Variant caller**	**15000X**					**20000X**					**25000X**				
	**NC**	**TP**	**FP**	**S(%)**	**P(%)**	**NC**	**TP**	**FP**	**S(%)**	**P(%)**	**NC**	**TP**	**FP**	**S(%)**	**P(%)**
DeepSNVMiner	44	44	0	88.00	100.00	44	44	0	88.00	100.00	43	43	0	86.00	100.00
MAGERI	41	41	0	82.00	100.00	41	41	0	82.00	100.00	41	41	0	82.00	100.00
smCounter2	0	0	0	0.00	0.00	0	0	0	0.00	0.00	0	0	0	0.00	0.00
UMI-VarCal	48	48	0	96.00	100.00	48	48	0	96.00	100.00	46	46	0	92.00	100.00
SiNVICT	5	0	5	0.00	0.00	7	0	7	0.00	0.00	9	0	9	0.00	0.00
outLyzer	2	0	2	0.00	0.00	2	0	2	0.00	0.00	2	0	2	0.00	0.00
Pisces	13	0	13	0.00	0.00	14	0	14	0.00	0.00	14	0	14	0.00	0.00
LoFreq	77	10	67	20.00	12.99	82	8	74	16.00	9.76	94	10	84	20.00	10.64

NC: number of calls; NM: number of mutations; TP: true positive; FP: false positive; S: sensitivity; P: precision.

Table S8. Performance of eight low-frequency variant callers on simulated datasets with different sequencing depths (1000X, 5000X, 10000X, 15000X, 20000X, and 25000X) at a VAF of 0.25%.

Variant caller	1000X					5000X					10000X				
	NC	TP	FP	S(%)	P(%)	NC	TP	FP	S(%)	P(%)	NC	TP	FP	S(%)	P(%)
DeepSNVMiner	0	0	0	0.00	0.00	43	43	0	86.00	100.00	44	44	0	88.00	100.00
MAGERI	2	0	2	0.00	0.00	40	40	0	80.00	100.00	41	41	0	82.00	100.00
smCounter2	0	0	0	0.00	0.00	0	0	0	0.00	0.00	0	0	0	0.00	0.00
UMI-VarCal	0	0	0	0.00	0.00	48	48	0	96.00	100.00	47	47	0	94.00	100.00
SiNVICT	0	0	0	0.00	0.00	2	0	2	0.00	0.00	3	0	3	0.00	0.00
outLyzer	0	0	0	0.00	0.00	1	0	1	0.00	0.00	1	0	1	0.00	0.00
Pisces	1	0	1	0.00	0.00	8	0	8	0.00	0.00	11	0	11	0.00	0.00
LoFreq	4	0	4	0.00	0.00	24	0	24	0.00	0.00	57	1	56	2.00	1.75

Variant caller	15000X					20000X					25000X				
	NC	TP	FP	S(%)	P(%)	NC	TP	FP	S(%)	P(%)	NC	TP	FP	S(%)	P(%)
DeepSNVMiner	44	44	0	88.00	100.00	44	44	0	88.00	100.00	43	43	0	86.00	100.00
MAGERI	41	41	0	82.00	100.00	40	40	0	80.00	100.00	41	41	0	82.00	100.00
smCounter2	0	0	0	0.00	0.00	0	0	0	0.00	0.00	0	0	0	0.00	0.00
UMI-VarCal	48	48	0	96.00	100.00	47	47	0	94.00	100.00	46	46	0	92.00	100.00
SiNVICT	5	0	5	0.00	0.00	8	0	8	0.00	0.00	9	0	9	0.00	0.00
outLyzer	2	0	2	0.00	0.00	2	0	2	0.00	0.00	2	0	2	0.00	0.00
Pisces	13	0	13	0.00	0.00	14	0	14	0.00	0.00	14	0	14	0.00	0.00
LoFreq	69	3	66	6.00	4.35	72	3	69	6.00	4.17	85	3	82	6.00	3.53

NC: number of calls; NM: number of mutations; TP: true positive; FP: false positive; S: sensitivity; P: precision.

Table S9. Performance of eight low-frequency variant callers on simulated datasets with different sequencing depths (1000X, 5000X, 10000X, 15000X, 20000X, and 25000X) at a VAF of 0.1%.

Variant caller	1000X					5000X					10000X				
	NC	TP	FP	S(%)	P(%)	NC	TP	FP	S(%)	P(%)	NC	TP	FP	S(%)	P(%)
DeepSNVMiner	0	0	0	0.00	0.00	0	0	0	0.00	0.00	43	43	0	86.00	100.00
MAGERI	2	0	2	0.00	0.00	0	0	0	0.00	0.00	38	38	0	76.00	100.00
smCounter2	0	0	0	0.00	0.00	0	0	0	0.00	0.00	0	0	0	0.00	0.00
UMI-VarCal	0	0	0	0.00	0.00	0	0	0	0.00	0.00	46	46	0	92.00	100.00
SiNVICT	0	0	0	0.00	0.00	1	0	1	0.00	0.00	4	0	4	0.00	0.00
outLyzer	0	0	0	0.00	0.00	1	0	1	0.00	0.00	1	0	1	0.00	0.00
Pisces	1	0	1	0.00	0.00	8	0	8	0.00	0.00	11	0	11	0.00	0.00
LoFreq	4	0	4	0.00	0.00	23	0	23	0.00	0.00	44	0	44	0.00	0.00

Variant caller	15000X					20000X					25000X				
	NC	TP	FP	S(%)	P(%)	NC	TP	FP	S(%)	P(%)	NC	TP	FP	S(%)	P(%)
DeepSNVMiner	44	44	0	88.00	100.00	43	43	0	86.00	100.00	44	44	0	88.00	100.00
MAGERI	41	41	0	82.00	100.00	39	39	0	78.00	100.00	41	41	0	82.00	100.00
smCounter2	0	0	0	0.00	0.00	0	0	0	0.00	0.00	0	0	0	0.00	0.00
UMI-VarCal	46	46	0	92.00	100.00	46	46	0	92.00	100.00	44	44	0	88.00	100.00
SiNVICT	5	0	5	0.00	0.00	7	0	7	0.00	0.00	9	0	9	0.00	0.00
outLyzer	2	0	2	0.00	0.00	2	0	2	0.00	0.00	2	0	2	0.00	0.00
Pisces	13	0	13	0.00	0.00	14	0	14	0.00	0.00	14	0	14	0.00	0.00
LoFreq	61	0	61	0.00	0.00	83	0	83	0.00	0.00	83	0	83	0.00	0.00

NC: number of calls; NM: number of mutations; TP: true positive; FP: false positive; S: sensitivity; P: precision.

Table S10. Performance of eight low-frequency variant callers on simulated datasets with different sequencing depths (1000X, 5000X, 10000X, 15000X, 20000X, and 25000X) at a VAF of 0.05%.

Variant caller	1000X					5000X					10000X				
	NC	TP	FP	S(%)	P(%)	NC	TP	FP	S(%)	P(%)	NC	TP	FP	S(%)	P(%)
DeepSNVMiner	0	0	0	0.00	0.00	0	0	0	0.00	0.00	0	0	0	0.00	0.00
MAGERI	2	0	2	0.00	0.00	0	0	0	0.00	0.00	0	0	0	0.00	0.00
smCounter2	0	0	0	0.00	0.00	0	0	0	0.00	0.00	0	0	0	0.00	0.00
UMI-VarCal	0	0	0	0.00	0.00	0	0	0	0.00	0.00	0	0	0	0.00	0.00
SiNVICT	0	0	0	0.00	0.00	2	0	2	0.00	0.00	3	0	3	0.00	0.00
outLyzer	0	0	0	0.00	0.00	1	0	1	0.00	0.00	1	0	1	0.00	0.00
Pisces	1	0	1	0.00	0.00	8	0	8	0.00	0.00	10	0	10	0.00	0.00
LoFreq	6	0	6	0.00	0.00	26	0	26	0.00	0.00	45	0	45	0.00	0.00

Variant caller	15000X					20000X					25000X				
	NC	TP	FP	S(%)	P(%)	NC	TP	FP	S(%)	P(%)	NC	TP	FP	S(%)	P(%)
DeepSNVMiner	0	0	0	0.00	0.00	44	44	0	88.00	100.00	44	44	0	88.00	100.00
MAGERI	0	0	0	0.00	0.00	39	39	0	78.00	100.00	40	40	0	80.00	100.00
smCounter2	0	0	0	0.00	0.00	0	0	0	0.00	0.00	0	0	0	0.00	0.00
UMI-VarCal	0	0	0	0.00	0.00	42	42	0	84.00	100.00	40	40	0	80.00	100.00
SiNVICT	5	0	5	0.00	0.00	7	0	7	0.00	0.00	9	0	9	0.00	0.00
outLyzer	2	0	2	0.00	0.00	2	0	2	0.00	0.00	2	0	2	0.00	0.00
Pisces	13	0	13	0.00	0.00	14	0	14	0.00	0.00	14	0	14	0.00	0.00
LoFreq	71	0	71	0.00	0.00	76	0	76	0.00	0.00	82	0	82	0.00	0.00

NC: number of calls; NM: number of mutations; TP: true positive; FP: false positive; S: sensitivity; P: precision.

Table S11. Performance of eight low-frequency variant callers on simulated datasets with different sequencing depths (1000X, 5000X, 10000X, 15000X, 20000X, and 25000X) at a VAF of 0.025%.

Variant caller	1000X					5000X					10000X				
	NC	TP	FP	S(%)	P(%)	NC	TP	FP	S(%)	P(%)	NC	TP	FP	S(%)	P(%)
DeepSNVMiner	0	0	0	0.00	0.00	0	0	0	0.00	0.00	0	0	0	0.00	0.00
MAGERI	1	0	1	0.00	0.00	0	0	0	0.00	0.00	0	0	0	0.00	0.00
smCounter2	0	0	0	0.00	0.00	0	0	0	0.00	0.00	0	0	0	0.00	0.00
UMI-VarCal	0	0	0	0.00	0.00	0	0	0	0.00	0.00	0	0	0	0.00	0.00
SiNVICT	0	0	0	0.00	0.00	1	0	1	0.00	0.00	4	0	4	0.00	0.00
outLyzer	0	0	0	0.00	0.00	1	0	1	0.00	0.00	1	0	1	0.00	0.00
Pisces	1	0	1	0.00	0.00	9	0	9	0.00	0.00	10	0	10	0.00	0.00
LoFreq	6	0	6	0.00	0.00	25	0	25	0.00	0.00	44	0	44	0.00	0.00

Variant caller	15000X					20000X					25000X				
	NC	TP	FP	S(%)	P(%)	NC	TP	FP	S(%)	P(%)	NC	TP	FP	S(%)	P(%)
DeepSNVMiner	0	0	0	0.00	0.00	0	0	0	0.00	0.00	0	0	0	0.00	0.00
MAGERI	0	0	0	0.00	0.00	0	0	0	0.00	0.00	0	0	0	0.00	0.00
smCounter2	0	0	0	0.00	0.00	0	0	0	0.00	0.00	1	0	1	0.00	0.00
UMI-VarCal	0	0	0	0.00	0.00	0	0	0	0.00	0.00	0	0	0	0.00	0.00
SiNVICT	5	0	5	0.00	0.00	7	0	7	0.00	0.00	9	0	9	0.00	0.00
outLyzer	2	0	2	0.00	0.00	2	0	2	0.00	0.00	2	0	2	0.00	0.00
Pisces	13	0	13	0.00	0.00	14	0	14	0.00	0.00	14	0	14	0.00	0.00
LoFreq	66	0	66	0.00	0.00	80	0	80	0.00	0.00	83	0	83	0.00	0.00

NC: number of calls; NM: number of mutations; TP: true positive; FP: false positive; S: sensitivity; P: precision.

Table S12. The execution time of each low-frequency variant caller.

Variant caller	5000X	10000X	15000X	20000X	25000X
DeepSNVMiner	185	278	380	473	556
smCounter2	739	1615	2524	3419	4302
UMI-VarCal	51	96	145	187	232
MAGERI	11	14	19	22	28
SiNVICT	82	160	323	320	394
outLyzer	84	114	141	172	196
Pisces	76	141	208	274	341
LoFreq	161	393	692	1061	1466

The execution time was measured in seconds.

Supplementary Figures

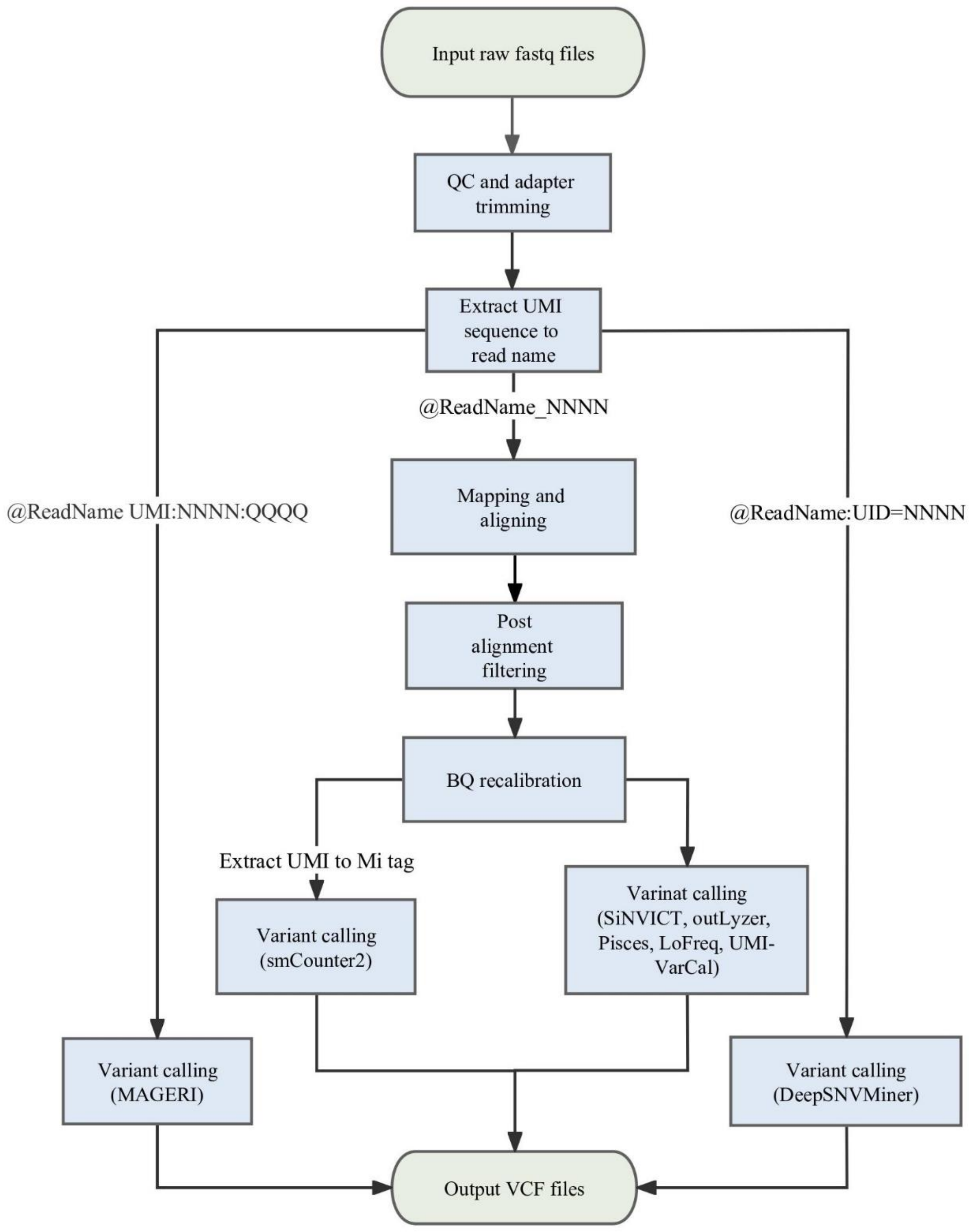

Figure S1. The flowchart of benchmarking analysis of eight variant calling tools.
